# A new recipe for TARTs? One step closer in identifying the origin of testicular adrenal rest tumours

**DOI:** 10.1530/EJE-22-0784

**Published:** 2022-09-26

**Authors:** Leonardo Guasti, James F H Pittaway

**Affiliations:** 1 Centre for Endocrinology, William Harvey Research Institute, Barts and the London Faculty of Medicine and Dentistry, Queen Mary University of London, London, UK

Testicular adrenal rest tumours (TARTs) are non-malignant lesions that occur in male patients exposed to high levels of adrenocorticotropic hormone (ACTH), especially when this occurs *in utero* or in early childhood. This is seen most commonly in the context of congenital adrenal hyperplasia (CAH) but can also occur in Cushing’s disease and is reported in Nelson’s syndrome and Addison’s disease. It is in CAH that TARTs are most prominent in clinical practice where they are present in approximately 40% of male patients (1).

The dogma has been that these tumours form from ‘adrenal rests’, cells of adrenal morphology and function that have occurred in the testis as a result of co-opted migration and separation of the adrenogonadal primordium. The proposition was that these cells may occur physiologically in the fetal and neonatal gonads but, without the stimulus of supraphysiological ACTH drive, regress spontaneously within the first year of life (1). This hypothesis was supported by the reported expression in TARTs of adrenal-specific genes such as 11-β hydroxylases 1 and 2 (*CYP11B1*, *CYP11B2*), 21-α hydroxylase 2 (*CYP21A2*), Delta Like Non-Canonical Notch Ligand 1 (*DLK1*) and the ACTH receptor (melanocortin 2 (*MC2R*) (2, 3).

However, it has become better delineated that these tumours also express testis-specific genes such as the luteinising hormone/choriogonadotrophin receptor (*LHCGR*), insulin-like peptide 3 (*INSL3*) and 17 β-hydroxysteroid dehydrogenase 3 (*HSD17B3*) (2). As such, it is recognised that TARTs likely originate from a more pluripotent steroidogenic cell type from earlier in fetal development. Identifying the true origin of these cells is an important step in better understanding the development of these tumours.

Claahsen-van der Grinten *et al.* (4) have proposed that the development of TARTs occurs in five stages. TART cells are present in the rete testis where they start to undergo hyperplasia and hypertrophy, at least partly under the influence of ACTH. As the growth continues, the lesion becomes fibrosed and starts to compress the rete testis. Inflammatory infiltration occurs as fibrosis worsens and this along with the growth of the tumour leads to irreversible damage to the testicular parenchyma. As this is commonly seen as a bilateral phenomenon (in 77% of patients), this can lead to subfertility and TARTs are the most common cause of infertility in CAH male patients (5).

Treatment of TARTs is focussed at restoring fertility. Surgical resection has not been proven conclusively to improve fertility and as such is only indicated in those who suffer from severe pain from the tumours. The mainstay of other treatments is medical and is focussed on controlling ACTH levels with glucocorticoids. However, this is not always successful and indeed TARTs also occur in CAH patients with well-controlled ACTH levels.

The other prominent clinical issue with TARTs is that they are difficult to distinguish clinically from Leydig cell tumours. Unlike TARTs, these tumours carry a 10% rate of malignant transformation and therefore are important to distinguish. Leydig cell tumours are rarely bilateral and not commonly seen in CAH, but it is an important distinction to make in the face of a unilateral testicular lesion in these patients (5). As such, there is a burgeoning interest in better defining the origin of TARTs, to improve the clinical care and outcomes of patients who are affected by them.

In a new study published in this journal, Schröder and colleagues have helped to further define the phenotype of TARTs and intriguingly shed some light on the possible cell of origin in these tumours (6). By performing double immunofluorescence in TART cells, the authors found that a proportion of TART cells co-expressed both 11-β hydroxylase (adrenal-specific and encoded by the *CYP11B1* gene) and 17β-Hydroxysteroid dehydrogenase 3 (Leydig cell-specific, encoded by the *HSD17B3* gene) indeed indicating the presence of ‘hybrid’ cells with an adreno-testicular phenotype, rather than a heterogeneous population of separate adrenal-like and testicular-like cells within the tumour parenchyma. However, they also detected cells that only expressed *CYP11B1*, as a population more consistent with an adrenal phenotype. These data are supported by transcriptomics that was carried out in which they showed the gene expression signature of TARTs is most similar to that of the adult adrenal followed by the adult testis and least similar to their embryonic counterparts. Additionally, TARTs did not express particularly high levels of fetal genes but corroborated evidence that they expressed both adrenal- and testis-specific genes.

They further showed that adult progenitor Leydig cells do not express MC2R. This means that if these cells are the origin of TARTs then their putative differentiation into steroidogenic adrenal cells is not through the action of ACTH on the MC2R receptor. However, cAMP/PKA activation was able to induce *CYP11B1* expression suggesting an alternate pathway or receptor may be responsible for this differentiation and leaving the door open for the candidacy of these cells to be the origin of TARTs (6).

This research supports the hypothesis that during embryonic development, a yet-to-be-identified bipotent adrenogonadal precursor could aberrantly respond to promoting factors (such as ACTH, angiotensin II or other) and then differentiate into TART cells retaining signatures of both adrenal and testis.

Development of TARTs has not been reported in mouse models recapitulating cardinal features of CAH, as they could be employed in genetic lineage tracing experiments aimed at fully uncovering the cell of origin of these tumours. In the absence of such models, future single-cell sequencing and spatial transcriptomics experiments in humans will further enhance our knowledge in the pathobiology of TARTs, including their cell of origin.

## Declaration of interest

The authors declare that there is no conflict of interest that could be perceived as prejudicing the impartiality of this commentary.

## Funding

L G and J F H P are supported from grants from the Medical Research Council http://dx.doi.org/10.13039/501100000265 (MRC, grant number MR/S022155/1), Biotechnology and Biological Sciences Research Council http://dx.doi.org/10.13039/501100000268 (BBSRC, grant number BB/V007246/1) and Barts Charity http://dx.doi.org/10.13039/100015652 (grant number MGU0436).
